# COVID-19 impact on index testing services and programmatic cost in 5 high HIV prevalence Indian districts

**DOI:** 10.1186/s12879-022-07912-3

**Published:** 2022-12-08

**Authors:** Rose Pollard, Ajay Enugu, Salin Sriudomporn, Jade Bell, Subash Chandra Ghosh, Visvanathan Arumugam, Parthasarathy Mugundu, Aditya Singh, Allison M. McFall, Shruti H. Mehta, Bryan N. Patenaude, Sunil S. Solomon

**Affiliations:** 1 Division of Infectious Diseases, The Johns Hopkins University School of Medicine, 1830 E. Monument St, Baltimore, MD 21205 USA; 2 International Vaccine Access Center, Johns Hopkins Bloomberg School of Public Health, 415 N Washington St, Baltimore, MD 21231 USA; 3 Y.R. Gaitonde Centre for AIDS Research and Education (YRG CARE), 58 Harrington Road, Chetput, Chennai, 600031 India; 4 Department of Epidemiology, The Johns Hopkins Bloomberg School of Public Health, 615 N Wolfe St, Baltimore, MD 21205 USA; 5 Department of International Health, The Johns Hopkins Bloomberg School of Public Health, 615 N Wolfe St, Baltimore, MD 21205 USA

**Keywords:** HIV, Cost analysis, Index testing, India

## Abstract

**Background:**

Restrictions to curb the first wave of COVID-19 in India resulted in a decline in facility-based HIV testing rates, likely contributing to increased HIV transmission and disease progression. The programmatic and economic impact of COVID-19 on index testing, a standardized contact tracing strategy, remains unknown.

**Methods:**

Retrospective programmatic and costing data were analyzed under a US government-supported program to assess the pandemic’s impact on the programmatic outcomes and cost of index testing implemented in two Indian states (Maharashtra and Andhra Pradesh). We compared index testing continuum outcomes during lockdown (April–June 2020) and post-lockdown (July–Sept 2020) relative to pre-lockdown (January–March 2020) by estimating adjusted rate ratios (aRRs) using negative binomial regression. Startup and recurrent programmatic costs were estimated across geographies using a micro-costing approach. Per unit costs were calculated for each index testing continuum outcome.

**Results:**

Pre-lockdown, 2431 index clients were offered services, 3858 contacts were elicited, 3191 contacts completed HIV testing, 858 contacts tested positive, and 695 contacts initiated ART. Compared to pre-lockdown, the number of contacts elicited decreased during lockdown (aRR = 0.13; 95% CI: 0.11–0.16) and post-lockdown (aRR = 0.49; 95% CI: 0.43–0.56); and the total contacts newly diagnosed with HIV also decreased during lockdown (aRR = 0.22; 95% CI: 0.18–0.26) and post-lockdown (aRR = 0.52; 95% CI: 0.45–0.59). HIV positivity increased from 27% pre-lockdown to 40% during lockdown and decreased to 26% post-lockdown. Further, ART initiation improved from 81% pre-lockdown to 88% during lockdown and post-lockdown. The overall cost to operate index testing was $193,457 pre-lockdown and decreased during lockdown to $132,177 (32%) and $126,155 (35%) post-lockdown. Post-lockdown unit cost of case identification rose in facility sites ($372) compared to pre-lockdown ($205), however it decreased in community-based sites from pre-lockdown ($277) to post-lockdown ($166).

**Conclusions:**

There was a dramatic decline in the number of index testing clients in the wake of COVID-19 restrictions that resulted in higher unit costs to deliver services; yet, improved linkage to ART suggests that decongesting centres could improve efficiency. Training index testing staff to provide support across services including non-facility-based HIV testing mechanisms (i.e., telemedicine, HIV self-testing, community-based approaches) may help optimize resources during public health emergencies.

**Supplementary Information:**

The online version contains supplementary material available at 10.1186/s12879-022-07912-3.

## Background

Innovative efforts are needed to maintain human immunodeficiency virus (HIV) testing services given barriers to facility-based testing which worsened over the coronavirus disease 2019 (COVID-19) pandemic, such as reduced access to health facilities and higher client volumes. To sustain gains towards HIV epidemic control, adaptions are especially needed to reach those at highest risk of HIV. Partner/contact testing (often referred to as index testing) is a critical strategy for identifying higher risk individuals and supporting them in getting tested for HIV. Index testing essentially represents a standardized process for HIV contact tracing. The goal of index testing is to elicit contacts of people living with HIV (PLHIV), including their spouses, sexual and needle sharing partners, and biological children, and offer support to those contacts for HIV testing and follow up. This includes support for rapid antiretroviral therapy (ART) initiation if a contact tests positive for HIV. The targeted strategy has been shown to increase the number of people tested for HIV and the number of PLHIV linked to care and treatment in low- and middle- income countries (LMIC), presenting an efficient approach to achieve The Joint United Nations Programme on HIV/AIDS (UNAIDS) 95-95-95 goals [1–3].

India reported its first COVID-19 case in January 2020 [4]. While case counts were still relatively low, the Indian Government imposed a national lockdown to help mitigate the spread of COVID-19. This lockdown was imposed at the end of March 2020 and extended until June 2020 [5]. The government started to ease national lockdown measures from July 2020 onward, however certain states still maintained restrictions in mobility and service delivery. Daily case counts increased again and by the end of September, there were over six million confirmed cases in India [6]. During the first wave lockdown period, all non-essential activities and transportation were suspended. Medical facilities had an influx of COVID-19 patients and many staff were reassigned to COVID-19 services, which disrupted the capacity of other health services [7]. The state of Maharashtra had the highest number of COVID-19 cases across India, with 14 hotspot areas designated as red zones where case counts were concentrated [8, 9]. In these hotspots, lockdowns were the most extreme which severely restricted people’s movement outside of their homes.

Restrictions and lockdowns following the emergence of COVID-19 in India caused significant disruptions to HIV services, as staff effort was redirected to COVID-19 patients and many facilities stopped offering HIV testing services during lockdown. These disturbances presented challenges for a program supported by the U.S. President’s Emergency Plan for AIDS Relief (PEPFAR) operating index testing in two Indian states, Maharashtra and Andhra Pradesh, to maintain service continuity over the pandemic. We evaluated the impact of COVID-19 from January to September 2020 on index testing programmatic outputs and operational costs in Maharashtra and Andhra Pradesh. Findings can inform HIV public health implementers consider strategies to maintain HIV testing services and linkage to care given the ongoing COVID-19 crisis and anticipated additional surges in India and other countries.

## Methods

### Program context

**Fig. 1:**
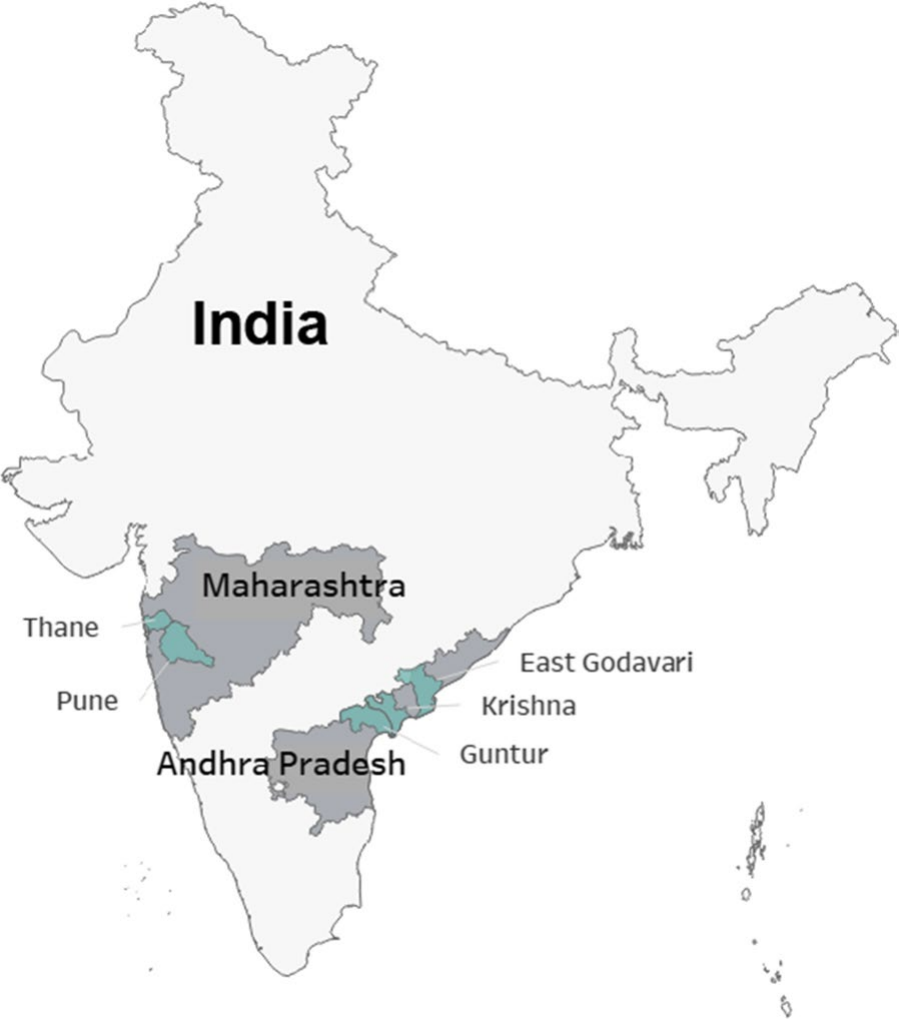
Geographies of index testing implementation

Under a PEPFAR-supported program that provides assistance to the national program to improve the HIV care continuum in India, we initiated index testing services in October 2019 in five high HIV prevalence districts in two Indian states (East Godavari, Guntur, and Krishna districts in Andhra Pradesh; Pune and Thane districts in Maharashtra) (Fig. 1). Index testing services were offered at 55 sites (48 facilities, seven community sites). Facility-based sites included public sector integrated counseling and testing centres (ICTCs) and ART centres. At testing centres, all clients who recently tested positive for HIV were offered index testing services. At ART centres, index testing services were offered to PLHIV who recently initiated treatment, recently re-initiated treatment, were not virally suppressed, or who reported sub-optimal adherence. In Andhra Pradesh, facility-based efforts were additionally supported by community-based index testing, which worked to reach clients unreached by facilities. Community sites were identified based on the current coverage of facilities as well as network mapping of PLHIV previously identified. At both facility and community-based sites, trained coordinators offered index testing services to clients, obtained consent, and worked with clients to elicit their contacts (spouses, sexual/needle-sharing partners, biological children) who may have been exposed to HIV. A notification plan was determined for each contact, in collaboration with and with agreement of the client. Screening for intimate partner violence (and linkage to support services, if needed) was carried out with clients prior to partner notification. Index testing coordinators followed up with either the client or the contact, depending on the agreed upon notification plan, to support contacts’ linkage to HIV testing services. Linkage to HIV testing sometimes required months of follow-up from index testing coordinators, especially as COVID-19 restrictions intensified. HIV testing was provided by the National AIDS Control Programme of India.

### Data analysis

To evaluate the programmatic and economic impact of COVID-19 on index testing services, we analyzed data across three periods: pre-lockdown (Jan–Mar 2020), lockdown (Apr–Jun 2020), and post-lockdown (Jul–Sep 2020). We compared data from lockdown and post-lockdown periods to the pre-lockdown period. Outcomes were measured along the index testing continuum using programmatic data captured through routine index testing activities and monthly expenditure data of the program implementing index testing. Index testing continuum outcomes included (1) number of clients offered index testing services; (2) number of clients who accepted index testing services; (3) number of contacts elicited; (4) number of contacts tested for HIV; (5) number of contacts who tested HIV positive; (6) number who initiated ART; (7) time in days to ART initiation among newly diagnosed contacts. We also examined the average number of contacts elicited per index client, proportion of contacts who tested positive for HIV, and proportion of newly diagnosed contacts who initiated ART. Outcomes were stratified by time period, state, and implementation category (facility-based and community-based). The chi-square test was used to test for significance between variables. Negative binomial regression was used to estimate age and gender adjusted rate ratios (aRRs), comparing the counts of total index testing clients, contacts elicited, contacts tested, newly diagnosed contacts, and contacts initiated on ART across the three periods.

The costs of operating the program during all three periods were captured through direct project expenditure data during the corresponding period. Startup and recurrent programmatic costs were calculated using a micro-costing approach [10]. Cost inputs included personnel, travel and transportation, training, supplies and equipment, program management and other operational costs (Additional file 1). Districts where these costs were incurred were identified and costs were allocated accordingly. Costs that did not have a location identified were allocated equally among all districts where the activity was executed. Because the aim was to estimate the cost of operating index testing services under the PEPFAR program, the costs for HIV testing kits and ART medication supplied by India’s National AIDS Control Programme were not included in the analysis. Overall activity costs were calculated, along with cost per individual, across the index testing continuum outcomes. All costs are in 2019 United States dollars (USD).

## Results

### Index testing programmatic outputs

From January to September 2020, 3109 index clients participated in index testing services (58% men, 42% women, 0.5% transgender individuals) (Table 1 presents outcomes per individual by time period. Client and contact characteristics can be found in Additional file 2.) From participating clients, 6228 contacts were elicited (47% men, 53% women, 0.6% transgender individuals) for an average of two contacts per index client. The gender distribution of index clients and contacts elicited remained relatively constant across the three time periods of analysis. Of the 5398 contacts who completed HIV testing across all time periods, 1394 (26%) were spouses of index clients, 3396 (63%) were sexual partners other than spouses, and 608 (11%) were children of index clients.

**Table 1:** Outcomes and cost per individual across index testing cascade by time period (costs in USD)

	Total		Pre-lockdown		Lockdown		Post-lockdown	
	n	Cost per unit	n	Cost per unit	n	Cost per unit	n	Cost per unit
Index clients offered services	3318	$136	2431	$80	179	$738	708	$178
Facility	2855	$114	2119	$59	151	$712	585	$162
Community	463	$271	312	$222	28	$881	123	$254
Index clients accepted services	3109	$145	2258	$86	171	$773	680	$186
Facility	2646	$123	1946	$64	143	$752	557	$170
Community	463	$271	312	$222	28	$881	123	$254
Contacts elicited	6228	$73	3858	$50	504	$262	1866	$68
Facility	4319	$76	2847	$44	302	$356	1170	$81
Community	1909	$66	1011	$69	202	$122	696	$45
Contacts completed HIV testing	5398	$84	3191	$61	500	$264	1707	$74
Facility	3498	$93	2180	$57	298	$361	1020	$93
Community	1900	$66	1011	$69	202	$122	687	$46
Contacts tested positive	1499	$301	858	$225	198	$668	443	$285
Facility	969	$337	605	$205	109	$986	255	$372
Community	531	$236	254	$273	89	$277	188	$166
Contacts initiated on ART	1,258	$359	695	$278	174	$760	389	$324
Facility	803	$407	491	$253	95	$1132	217	$437
Community	455	$275	204	$340	79	$312	172	$182

**Fig. 2:**
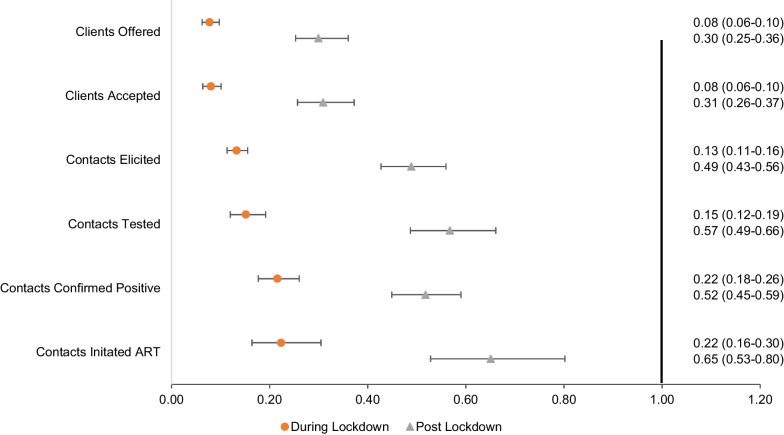
Adjusted rate ratios for index testing outcomes by time period. The pre-lockdown period was used as the reference period

**Fig. 3:**
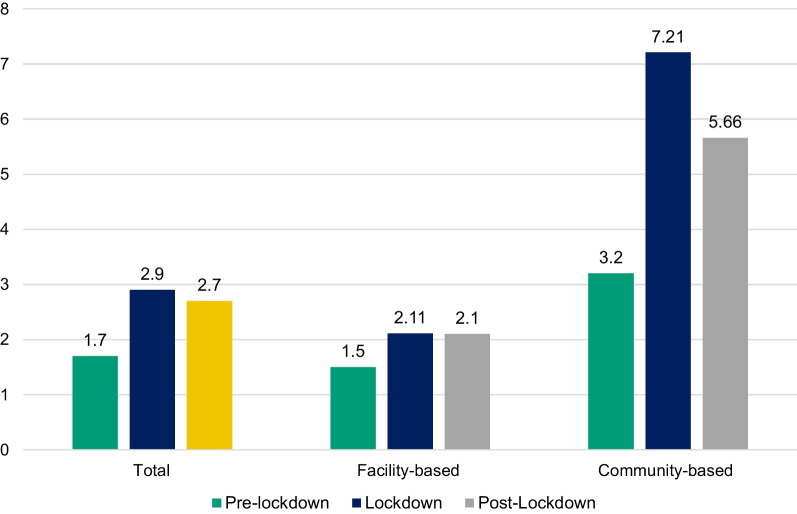
Average number of contacts elicited per index client across time periods by activity modality

Over the course of the pandemic, statistically significant declines were observed in the number of index clients who were offered services which in turn was associated with statistically significant reductions in contacts elicited. The number of contacts elicited from index clients declined almost 90% (aRR = 0.13; 95% confidence interval (CI): 0.11–0.16) (Fig. 2, Additional file 3) from 3858 pre-lockdown to 504 during lockdown. While contact elicitation in the post-lockdown period improved, the number of contacts elicited remained significantly lower than the pre-pandemic period (aRR = 0.49; 95% CI: 0.43–0.56). The reductions in contacts elicited also resulted in significantly lower number of contacts tested during the lockdown and post-lockdown periods (p-values < 0.05 for both periods vs. pre-lockdown). Contrastingly, the average number of contacts elicited per index increased from 1.7 in the pre-pandemic period to 2.9 and 2.7 in the lockdown and post-lockdown periods, respectively (Fig. 3).

**Fig. 4:**
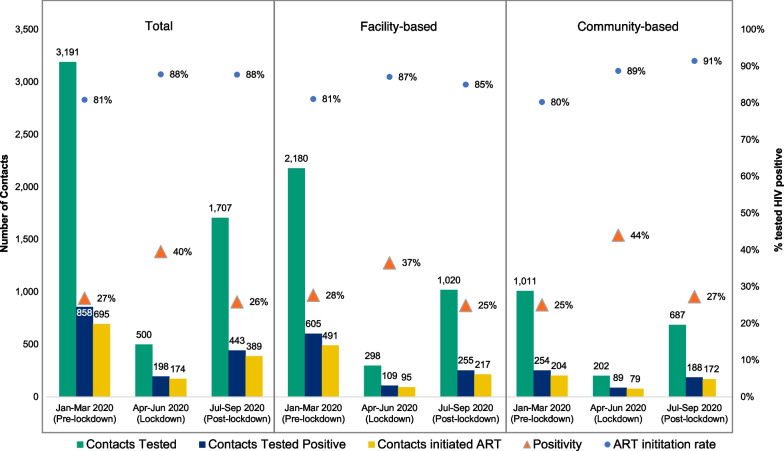
Outcomes of contact testing across time periods by activity modality

The total number of contacts who tested positive for HIV decreased from 858 pre-lockdown to 198 over lockdown (aRR = 0.22; 95% CI: 0.18–0.26) and to 443 post-lockdown (aRR = 0.52; 95% CI: 0.45–0.59). However, the HIV test positivity of contacts across all sites increased from 27% pre-lockdown to 40% in the lockdown period and returned to a similar level post-lockdown at 26% (Fig. 4). This increase was more pronounced in the community-based sites in Andhra Pradesh where test positivity increased from 25% during pre-lockdown to 44% during lockdown, compared to facility-based index testing (28% pre-lockdown, 37% lockdown).

The total number of newly diagnosed PLHIV initiated on ART decreased during lockdown (aRR 0.22; 95% CI: 0.16–0.30) and post-lockdown (aRR = 0.65; 95% CI: 0.53–0.80) compared to the pre-pandemic period. However, the proportion of newly diagnosed contacts who were linked to ART actually improved from 81% during the pre-pandemic period to 88% during the lockdown (p ≤ 0.001). Compared to men, women were significantly less likely to initiate ART post-lockdown (aRR = 0.72; 95% CI: 0.54–0.97) vs. pre-lockdown when controlling for age and time period. Community-based sites experienced a greater increase in ART initiation from pre-lockdown to post-lockdown (80–91%) compared to facility-based sites (81–85%). The average number of days from diagnosis to ART initiation decreased during lockdown as well as post-lockdown. Pre-lockdown, the average number of days to ART initiation across all sites was 8.1, which decreased to 7.3 over lockdown and 4.4. post-lockdown. This decline in number of days to ART initiation was similar in both facility and community sites (facility: 9.0 pre-lockdown, 6.2 lockdown, and 4.2 post-lockdown; community: 7.4 pre-lockdown, 5.4 lockdown, 4.9 post-lockdown).

### Index testing operational costs

The overall cost of operating index testing services decreased during COVID-19 lockdown and post-lockdown periods by 32% and 35% respectively, compared to pre-lockdown (pre-lockdown: $193,457, lockdown: $132,177, post-lockdown: $126,155). Labor costs continued to account for the majority of all expenditures for index testing services (pre-lockdown: 84%, lockdown: 95%, post-lockdown: 92%). Labor costs among community sites were notably lower than facility sites by 42.5% during pre-lockdown, by 79.9% during lockdown and by 70.8% post-lockdown. The higher operating cost among facility sites became more pronounced during lockdown and post-lockdown at 176% and 90% higher than community sites ($68,190 vs $24,671 during lockdown and $59,425 vs $31,279 during post-lockdown). In Andhra Pradesh, the costs of operating index testing among community sites were similar across operating districts, ranging by 6% and 4% for pre- and post-lockdown, respectively.

In facilities, the average cost to offer services to one index testing client increased from $59 pre-lockdown to $712 over lockdown and decreased to $162 post-lockdown (Table 1, Additional file 4). In community sites, the cost to offer services to one index testing client remained higher than facilities at $222 pre-lockdown, $881 over lockdown and $254 post-lockdown. In facilities pre-lockdown, the average cost to identify one HIV positive contact was $205 and to initiate a newly diagnosed contact on ART was $253. These facility-based costs increased during lockdown ($986 to identify one HIV positive contact and $1132 to initiate treatment) and reduced post-lockdown ($372 to identify one HIV positive contact and $437 to initiate treatment). In community sites, the average cost to identify one HIV positive contact was $273 pre-lockdown. This increased over the lockdown period to $277, but then decreased to $166, which was lower than pre-pandemic. The cost to initiate a newly diagnosed contact on ART in community settings also decreased from $340 pre-lockdown to $312 over lockdown and decreased further to $182 post-lockdown.

## Discussion

These data demonstrate the programmatic and economic implications of COVID-19 on an active HIV testing program in two of the highest-burden Indian states. We observed a dramatic decline in the number of clients who were offered index testing services following lockdown restrictions which led to fewer index clients participating. The reduced number of index clients resulted in declines across the rest of the index testing cascade outcomes over the pandemic. This cumulatively resulted in a decline in the identification of new HIV diagnoses via index testing by as much as 97% during the lockdown period in the state of Maharashtra, the state with the most severe COVID-19 outbreak across India. Such a considerable drop in testing suggests ongoing transmission and disease progression, because fewer people were diagnosed and started on treatment. Contrastingly, the ratio of contacts elicited per index client as well as the number of contacts tested and linked to ART (including time from diagnosis to ART initiation) improved during the pandemic associated periods. Costs associated with diagnosis and initiation of ART increased significantly over lockdown. Although costs decreased post-lockdown, they remained higher than before the pandemic. The exception was community-based sites, which saw improved cost efficiency for diagnosis and ART initiation costs post-lockdown compared to pre-lockdown.

Our study adds to the evidence establishing partner testing as a feasible approach to achieve a high level of HIV case identification in LMICs even in settings with generalized HIV epidemics, [1–3, 11] and highlights the significant impacts of the COVID-19 pandemic on HIV testing services. Programmatic findings reflect some recovery in the post-lockdown period but case finding results across the cascade only returned to around 50% of what was seen in the pre-pandemic period. During the COVID-19 lockdown period, there was a dramatic decline in the number of participating index clients as well as contacts tested for HIV. This is in line with the significant reductions seen across India in facility-based HIV testing service access during the first wave of COVID-19, and mirrors a global trend in decreased HIV testing over COVID-19 lockdowns [12–15]. These declines reflect restricted access to in-person clinical and laboratory services during the pandemic [12]. However, in some countries including Mali, Brazil, United States, and China, HIV testing access and case finding rates were sustained over COVID-19 by expanding non-facility-based channels for testing, particularly HIV self-testing [16–19]. This highlights the potential of decentralized HIV testing modalities to overcome facility-based testing disruptions shown in our study, especially linkage gaps between contact elicitation and HIV screening.

Despite the drop in HIV testing numbers, our program saw an increase in the ratio of contacts elicited per index client over the pandemic, which could be explained by staff having more time for contact elicitation, resulting in higher numbers of contacts elicited per index. During lockdown and post-lockdown, our index to contact ratio was higher than what has been documented in previous index testing studies [20, 21]. The proportion of contacts who tested positive among those who completed testing also increased significantly over lockdown, which could be explained by the COVID-19 lockdown restrictions leading to only those at highest risk or the sickest coming in for testing. During this lockdown period, our HIV test positivity was higher or comparable to what has been shown in other studies documenting index testing outcomes in Sub-Saharan Africa [2, 3, 20, 22–24]. A 2017 study in Vietnam where the HIV epidemic is concentrated among key populations, similar to India, documented a high HIV positivity rate (42%) among partners of key populations tested for HIV through index testing, which is comparable to the rate seen over lockdown in our program [25].

Among those who tested positive, we saw improved linkage to ART initiation over the pandemic. It is possible that these clients were more motivated to access ART from a heightened consideration about health over the COVID-19 pandemic. Improved ART linkage may also be attributable to decreased patient load at ART centres because of lockdowns or the proactive measures taken by the National AIDS Control Organization to help ensure that PLHIV received uninterrupted ART supply over the pandemic. Measures included scaling up multi-month dispensation, home and community delivery of ART, and allowing clients to access ART at any facility, instead of only the one they are registered at [26]. These efforts contributed to decentralizing ART access and decreasing client loads at facilities, on top of the already diminished client flow due to COVID-19-related restrictions in movement.

With fewer clients showing up to facilities, staff likely had more time to spend with each client and to build relationships, which could have contributed to the improved efficiency of both contact elicitation and ART initiation seen in our program. Positive, reliable relationships with providers and case management services have in fact been shown to be pivotal in client outcomes and retention in HIV services [27, 28]. Further, client retention and adherence are critical determinants of program cost effectiveness, as sub-optimal ART adherence can lead to higher healthcare costs due to poorer health outcomes [29]. Therefore investing in ways to decentralize and decongest HIV services may improve HIV outcomes by optimizing index testing and ART centre staff efforts. In particular, expanding service approaches such as HIV self-testing and telemedicine, which give clients the flexibility to be in any location they choose rather than being required to be present in a physical facility, may reduce the number of clients who seek services in person at health centres. Consequently, this could give staff more time to spend with clients who are in person at facilities to help build relationships and improve client outcomes.

The increase in programmatic costs in our study relate to the decreased number of clients and contacts who completed HIV testing over the pandemic. Compared to data from a 2015 systematic review of HIV testing costs, our pre-lockdown unit cost to identify a new contact living with HIV was higher than the median cost of case identification from combined HIV testing strategies for general populations in LMICs ($113), but lower than the median cost for key populations ($324) [30]. Post-lockdown unit costs of case identification and ART initiation almost doubled in facility-based index testing compared to pre-lockdown. This reflected the decreased number of index clients recruited and fewer contacts elicited, compounded with decreased HIV testing rates during COVID-19. Because our labor costs remained consistent over COVID-19, the cost per client outcome increased. Despite this, decreasing the number of index testing staff to reduce labor costs over periods of decreased client flow (such as the lockdown period) would likely not result in decreased overall cost. The amount of time and resources to train index testing staff is significant, and hiring and training new staff when client flows increase again would prove inefficient and expensive.

Further, the experience of staff implementing the PEPFAR-supported program demonstrate that fostering consistent relationships between index testing staff and clients/contacts supports successful HIV testing and ART initiation. Letting go and hiring new staff would disrupt this relationship-based process. Instead, programs should train index testing staff to serve as multipurpose health workers over periods of reduced client flow to preserve program’s return on investment during public health emergencies by maintaining HIV outcomes along the cascade. Staff can contribute in other ways than their usual facility-based index testing duties, such as counseling clients remotely, facilitating HIV self-testing, or delivering services and commodities at community sites. An intervention in Zambia called CIRKUITS adopted this approach at the start of the pandemic [31]. Community health workers rapidly adapted by going to hotspot areas and community sites to conduct index testing services and distribute HIV self-test kits to partners. The intervention found that the number of clients tested for HIV and positivity yield remained stable over the first year of the pandemic, demonstrating the utility of this model. Accordingly, innovative strategies to re-imagine and adapt staff roles can support resiliency during phases of acute pandemic restrictions [32].

Our findings support the expansion of community-based index testing services for optimized case identification. The unit cost of case identification as well as ART initiation per individual decreased by half in community-based index testing from pre-lockdown to post-lockdown. Our post-lockdown unit cost for case identification in facilities rose above the global medians across all HIV testing strategies as reported in 2015, however in community sites, the post-lockdown unit cost of case identification dropped below global median costs for key populations as well as general populations [30]. These findings demonstrate that community-based approaches may present a strategic opportunity to optimize program costs while improving outcomes of HIV contact tracing, partner notification, and follow-up efforts, especially as COVID-19 impacts continue worldwide.

### Limitations

Our findings come with limitations. Data collected by the PEPFAR-supported program are not inclusive of all national program data in the cluster districts. This reduces the generalizability of findings, which should consider the HIV burden of each state and the extent of COVID-19 impact. There was also limited information captured through our index testing services surrounding individual-level characteristics beyond age and gender, which could help better understand who was being missed by services—for example, characteristics of clients who refused index testing. Expenditures reported in our study only reflect the cost of operating the activity under the particular PEPFAR program, and do not reflect all expenditures involved for complete implementation of index testing across the health system. For example, the costs of HIV testing kits were covered by the Government of India and thus not included in our costing analysis model. While a focus on programmatic expenditures only limits generalizability, our study provides relevant comparisons of efficiency in a PEPFAR programmatic context across facility-based and community-based programs during COVID-19 and still offers important insights into delivery strategies that optimize efficiencies and resilience in times of hardship. Further, the data used for this analysis is only inclusive of India’s first wave of COVID-19, which does not encompass the impact of subsequent waves of the pandemic. Despite these limitations, our study offers rare insight into the cost of implementing index testing with the context of programmatic outcomes.

## Conclusions

The significant declines in HIV testing service access seen in our index testing program, as well as globally, over the COVID-19 pandemic call for a paradigm shift in HIV testing modalities which bring testing closer to communities through HIV self-testing and community-based approaches. The cost efficiency achieved through community-based index testing compared to facility-based index testing in our study further supports the value of community-based HIV services. The increased costs to operate index testing services over COVID-19 lockdowns call for diversifying index testing staff capacity to maintain HIV services in other ways when public health services are disrupted, including telemedicine for remote counseling, HIV self-testing distribution, and service delivery at community sites. Such innovations can work to optimize resources and funding sources to maintain HIV prevention services and ensure continued access to HIV care over public health emergencies, especially for HIV epidemics concentrated among key populations.

## Supplementary Information

**Additional file 1: **Costing categories.

**Additional file 2: **Client and contact characteristics by time period.

**Additional file 3: **Unadjusted and adjusted rate ratios for index testing outcomes by time period.

**Additional file 4: **Cost per individual across index testing cascade by time period and state.

## Data Availability

The data that support the findings of this study are not publicly available. De-identified, aggregate data was used by the study team for analysis which are available from the corresponding author upon reasonable request.

## Abbreviations

aRR: Adjusted rate ratio
ART: Antiretroviral therapy
CI: Confidence interval
COVID-19: Coronavirus disease 2019
HIV: Human immunodeficiency virus
ICTC: Integrated counseling and testing centre
LMIC: Low- and middle- income countries
PEPFAR: President’s Emergency Plan for AIDS Relief
PLHIV: People living with HIV
UNAIDS: The Joint United Nations Programme on HIV/AIDS
USD: United States dollars
