# Correction: COVID-19 impact on index testing services and programmatic cost in 5 high HIV prevalence Indian districts

**DOI:** 10.1186/s12879-022-07949-4

**Published:** 2022-12-22

**Authors:** Rose Pollard, Ajay Enugu, Salin Sriudomporn, Jade Bell, Subash Chandra Ghosh, Visvanathan Arumugam, Parthasarathy Mugundu, Aditya Singh, Allison M. McFall, Shruti H. Mehta, Bryan N. Patenaude, Sunil S. Solomon

**Affiliations:** 1 Division of Infectious Diseases, The Johns Hopkins University School of Medicine, 1830 E. Monument St, Baltimore, MD 21205 USA; 2 International Vaccine Access Center, Johns Hopkins Bloomberg School of Public Health, 415 N Washington St, Baltimore, MD 21231 USA; 3 Y.R. Gaitonde Centre for AIDS Research and Education (YRG CARE), 58 Harrington Road, Chetput, Chennai 600031 India; 4 Department of Epidemiology, The Johns Hopkins Bloomberg School of Public Health, 615 N Wolfe St, Baltimore, MD 21205 USA; 5 Department of International Health, The Johns Hopkins Bloomberg School of Public Health, 615 N Wolfe St, Baltimore, MD 21205 USA

**Correction: BMC Infect Dis (2022) 22:918** **https://doi.org/10.1186/s12879-022-07912-3**

Following publication of the original article [1], the authors reported a production error. The incorrect version of Figure 4 was published, which omits the value “858” in the first blue bar. The corrected Fig. 4. Is supplied in this correction article and the original article [1] has been corrected.

**Fig. 4:**
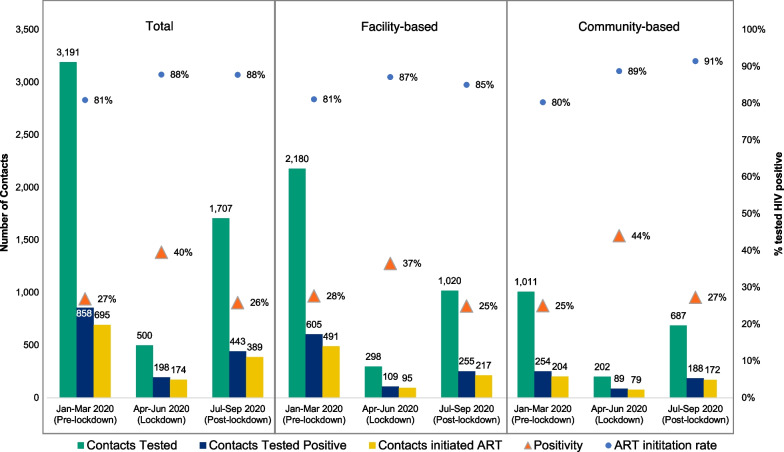
Outcomes of contact testing across time periods by activity modality
