# Supplementary material for: COVID-19 impact on index testing services and programmatic cost in 5 high HIV prevalence Indian districts
Source: BMC Infect Dis. 2022 Dec 8;22:918. doi: 10.1186/s12879-022-07912-3 (PMC9733361; doi:10.1186/s12879-022-07912-3)
Supplement: Supplementary file 1 — Additional file 1: Costing categories. [file 12879_2022_7912_MOESM1_ESM.pdf]

Additional File 1: Costing categories

**Fixed Costs**	**Recurrent Costs**
Start-up operational costs<br>- Initial community sensitization<br>- Advertisements	Operational costs<br>- Rent<br>- Utilities<br>- Communication (internet, wireless plans, staff phone etc.)
Trainings<br>- Venue, supplies, meals, staff and participant travel, trainers, lodging, per diem etc.	Trainings<br>- Venue, supplies, meals, staff and participant travel, trainers, lodging per diem, etc.
Supplies and Equipment<br>- Medical and non-medical supplies<br>- Site-level YRG offices (e.g. tablets, computers, furniture, vehicles etc.)<br>- Above-site JHU offices (e.g. tablets, computers, furniture, vehicles etc.)	Supplies and Equipment *excludes training*<br>- Medical and non-medical supplies<br>- Supplies for ongoing mobilization and sensitization<br>- Site-level YRG offices (e.g. tablets, computers, furniture, vehicles etc.)<br>- Above-site JHU offices (e.g. tablets, computers, furniture, vehicles etc.)
Construction: Not applicable<br>- Building/ Sites<br>- Refurbishment	Personnel (Salary &Fringe Benefits)<br>- Site-level YRG CARE staff<br>o Field-level services by staff type (e.g. outreach worker)<br>- Above-site staff<br>o YRG CARE and JHU Program Management staff<br>- Contractual/ Consultants
	Travel and Transportation *excludes training*<br>- Field mileage reimbursement, flights, lodging, per-diems for YRG CARE and JHU personnel
