# Supplementary material for: COVID-19 impact on index testing services and programmatic cost in 5 high HIV prevalence Indian districts
Source: BMC Infect Dis. 2022 Dec 8;22:918. doi: 10.1186/s12879-022-07912-3 (PMC9733361; doi:10.1186/s12879-022-07912-3)
Supplement: Supplementary file 2 — Additional file 2: Client and contact characteristics by time period. [file 12879_2022_7912_MOESM2_ESM.pdf]

Additional File 2: Client and contact characteristics by time period

		Total	Gender			Age			
			Men	Women	Trans-gender	1-14 Years	15-29 Years	30-44 Years	45+ Years
**Index clients offered services**	Pre-Lockdown Period	2,431	1,413 (58%)	1,007 (41%)	11 (0%)	1 (0%)	515 (21%)	1,227 (50%)	688 (28%)
	Lockdown Period	179	114 (64%)	64 (36%)	1 (1%)	0 (0%)	40 (22%)	80 (45%)	59 (33%)
	Post-Lockdown Period	708	388 (55%)	317 (45%)	3 (0%)	1 (0%)	170 (24%)	348 (49%)	189 (27%)
	**Total**	**3,318**	**1,915 (58%)**	**1,388 (42%)**	**15 (0%)**	**2 (0%)**	**725 (22%)**	**1,655 (50%)**	**936 (28%)**
**Index clients accepted services**	Pre-Lockdown Period	2,258	1,334 (59%)	916 (41%)	8 (0%)	1 (0%)	466 (21%)	1,148 (51%)	643 (28%)
	Lockdown Period	171	110 (64%)	60 (35%)	1 (1%)	0 (0%)	39 (23%)	76 (44%)	56 (33%)
	Post-Lockdown Period	680	372 (55%)	305 (45%)	3 (0%)	1 (0%)	150 (22%)	345 (51%)	184 (27%)
	**Total**	**3,109**	**1,816 (58%)**	**1,281 (41%)**	**12 (0%)**	**2 (0%)**	**655 (21%)**	**1,569 (50%)**	**883 (28%)**
**Contacts elicited**	Pre-Lockdown Period	3,858	1,805 (47%)	2,044 (53%)	9 (0%)	415 (11%)	1,143 (30%)	1,663 (43%)	637 (17%)
	Lockdown Period	504	222 (44%)	278 (55%)	4 (1%)	25 (5%)	145 (29%)	227 (45%)	107 (21%)
	Post-Lockdown Period	1,866	892 (48%)	952 (51%)	22 (1%)	164 (9%)	538 (29%)	847 (45%)	317 (17%)
	**Total**	**6,228**	**2,919 (47%)**	**3,274 (53%)**	**35 (1%)**	**604 (10%)**	**1,826 (30%)**	**2737 (44%)**	**1,061 (17%)**
**Contacts completed HIV testing**	Pre-Lockdown Period	3,191	1,518 (48%)	1,664 (52%)	9 (0%)	288 (9%)	991 (31%)	1,369 (43%)	543 (17%)
	Lockdown Period	500	223 (45%)	273 (55%)	4 (1%)	27 (5%)	147 (29%)	221 (44%)	107 (21%)
	Post-Lockdown Period	1,707	812 (48%)	865 (51%)	30 (2%)	120 (7%)	500 (29%)	788 (46%)	299 (18%)
	**Total**	**5,398**	**2,553 (47%)**	**2,802 (52%)**	**43 (1%)**	**435 (8%)**	**1,638 (30%)**	**2378 (44%)**	**949 (18%)**
**Contacts tested positive**	Pre-Lockdown Period	858	383 (45%)	474 (55%)	1 (0%)	25 (3%)	178 (21%)	424 (49%)	231 (27%)
	Lockdown Period	198	87 (44%)	111 (56%)	0 (0%)	0 (0%)	34 (17%)	105 (53%)	59 (30%)
	Post-Lockdown Period	443	227 (51%)	215 (49%)	1 (0%)	5 (1%)	83 (19%)	222 (50%)	133 (30%)
	**Total**	**1,499**	**697 (46%)**	**800 (53%)**	**2 (0%)**	**30 (2%)**	**295 (20%)**	**751 (50%)**	**423 (28%)**
**Contacts initiated on ART**	Pre-Lockdown Period	695	307 (44%)	388 (56%)	0 (0%)	18 (3%)	141 (20%)	344 (49%)	192 (28%)
	Lockdown Period	174	74 (43%)	100 (57%)	0 (0%)	1 (1%)	31 (18%)	89 (51%)	53 (30%)
	Post-Lockdown Period	389	208 (53%)	181 (47%)	0 (0%)	5 (1%)	69 (18%)	199 (51%)	116 (30%)
	**Total**	**1,258**	**589 (47%)**	**669 (53%)**	**0 (0%)**	**24 (2%)**	**241 (20%)**	**632 (50%)**	**361 (29%)**
