# Supplementary material for: COVID-19 impact on index testing services and programmatic cost in 5 high HIV prevalence Indian districts
Source: BMC Infect Dis. 2022 Dec 8;22:918. doi: 10.1186/s12879-022-07912-3 (PMC9733361; doi:10.1186/s12879-022-07912-3)
Supplement: Supplementary file 3 — Additional file 3: Unadjusted and adjusted rate ratios for index testing outcomes by time period. [file 12879_2022_7912_MOESM3_ESM.pdf]

Additional File 3: Unadjusted and adjusted rate ratios for index testing outcomes by time period
Adjusted variables were age and gender

			RR	(95% CI)	aRR	(95% CI)
**Index clients offered services**	Pre-Lockdown Period		ref.		ref.	
	Lockdown Period		0.08***	(0.06-0.10)	0.08***	(0.06-0.1)
	Post-Lockdown Period		0.29***	(0.23-0.37)	0.3***	(0.25-0.36)
**Index clients accepted services**	Pre-Lockdown Period		ref.		ref.	
	Lockdown Period		0.08***	(0.06-0.10)	0.08***	(0.06-0.10)
	Post-Lockdown Period		0.30***	(0.24-0.38)	0.31***	(0.26–0.37)
**Contacts elicited**	Pre-Lockdown Period		ref.		ref.	
	Lockdown Period		0.14***	(0.11-0.17)	0.13***	(0.11-0.16)
	Post-Lockdown Period		0.48***	(0.40-0.58)	0.49***	(0.43-0.56)
**Contacts completed HIV testing**	Pre-Lockdown Period		ref.		ref.	
	Lockdown Period		0.25***	(0.21-0.29)	0.15***	(0.12-0.19)
	Post-Lockdown Period		0.68***	(0.60-0.78)	0.57***	(0.49-0.66)
**Contacts tested positive**	Pre-Lockdown Period		ref.		ref.	
	Lockdown Period		0.27***	(0.22-0.34)	0.22***	(0.18-0.26)
	Post-Lockdown Period		0.59***	(0.50-0.70)	0.52***	(0.45-0.59)
**Contacts initiated on ART**	Pre-Lockdown Period		ref.		ref.	
	Lockdown Period		0.29***	(0.23-0.36)	0.22***	(0.16-0.3)
	Post-Lockdown Period		0.60***	(0.50-0.71)	0.65***	(0.53-0.8)
