# Supplementary material for: COVID-19 impact on index testing services and programmatic cost in 5 high HIV prevalence Indian districts
Source: BMC Infect Dis. 2022 Dec 8;22:918. doi: 10.1186/s12879-022-07912-3 (PMC9733361; doi:10.1186/s12879-022-07912-3)
Supplement: Supplementary file 4 — Additional file 4: Cost per individual across index testing cascade by time period and state. [file 12879_2022_7912_MOESM4_ESM.pdf]

Additional File 4: Cost per individual across index testing cascade by time period and state

	Total						Andhra Pradesh						Maharashtra					
	Pre-lockdown		Lockdown		Post-lockdown		Pre-lockdown		Lockdown		Post-lockdown		Pre-lockdown		Lockdown		Post-lockdown	
	n	Cost per unit	n	Cost per unit	n	Cost per unit	n	Cost per unit	n	Cost per unit	n	Cost per unit	n	Cost per unit	n	Cost per unit	n	Cost per unit
**Index clients offered services**	2,431	$80	179	$738	708	$178	966	$150	150	$619	456	$199	1,465	$33	29	$1,356	252	$141
Facility	2,119	$59	151	$712	585	$162	654	$115	122	$559	333	$178	1,465	$33	29	$1,356	252	$141
Community	312	$222	28	$881	123	$254	312	$222	28	$881	123	$254	0	NA	0	NA	0	NA
**Index clients accepted services**	2,258	$86	171	$773	680	$186	958	$151	148	$870	431	$299	1,300	$38	23	$1,541	249	$142
Facility	1,946	$64	143	$752	557	$170	646	$116	120	$495	308	$193	1,300	$38	23	$1,541	249	$142
Community	312	$222	28	$881	123	$254	312	$222	28	$2,479	123	$564	0	NA	0	NA	0	NA
**Contacts elicited**	3,858	$50	504	$262	1,866	$68	2,226	$65	480	$193	1,548	$59	1,632	$30	24	$1,638	318	$111
Facility	2,847	$44	302	$356	1,170	$81	1,215	$62	278	$245	852	$70	1,632	$30	24	$1,638	318	$111
Community	1,011	$69	202	$122	696	$45	1,011	$69	202	$122	696	$45	0	NA	0	NA	0	NA
**Contacts completed HIV testing**	3,191	$61	500	$264	1,707	$74	2,222	$65	475	$195	1,493	$61	969	$51	25	$1,573	214	$166
Facility	2,180	$57	298	$361	1,020	$93	1,211	$62	273	$250	806	$74	969	$51	25	$1,573	214	$166
Community	1,011	$69	202	$122	687	$46	1,011	$69	202	$122	687	$46	0	NA	0	NA	0	NA
**Contacts tested positive**	858	$225	198	$668	443	$285	634	$228	191	$486	393	$231	225	$218	7	$5,617	50	$709
Facility	605	$205	109	$986	255	$372	380	$198	102	$669	205	$290	225	$218	7	$5,617	50	$709
Community	254	$273	89	$277	188	$166	254	$273	89	$277	188	$166	0	NA	0	NA	0	NA
**Contacts initiated on ART**	695	$278	174	$760	389	$324	498	$290	167	$556	344	$264	197	$249	7	$5,617	45	$788
Facility	491	$253	95	$1,132	217	$437	294	$255	88	$775	172	$345	197	$249	7	$5,617	45	$788
Community	204	$340	79	$312	172	$182	204	$340	79	$312	172	$182	0	NA	0	NA	0	NA
